# Prevalence of antibiotic resistance in commensal *Escherichia coli* among the children in rural hill communities of Northeast India

**DOI:** 10.1371/journal.pone.0199179

**Published:** 2018-06-18

**Authors:** Ashish Kumar Singh, Saurav Das, Samer Singh, Varsha Rani Gajamer, Nilu Pradhan, Yangchen Doma Lepcha, Hare Krishna Tiwari

**Affiliations:** 1 Department of Microbiology, School of Life Sciences, Sikkim University, Gangtok, Sikkim, India; 2 Department of Microbial Biotechnology, Panjab University, Chandigarh, India; 3 State Institute of Rural Development, Government of Sikkim, Gangtok, Sikkim, India; Children's Hospital of Pittsburgh, University of Pittsburgh Medical Center, UNITED STATES

## Abstract

Commensal bacteria are the representative of the reservoir of antibiotic resistance genes present in a community. The usage of antibiotics along with the demographic factors is generally associated with an increase in antibiotics resistance in pathogens. Northeast (NE) India is untapped with regard to antibiotic resistance prevalence and spread. In the current study, the prevalence of antibiotic-resistant commensal *Escherichia coli* in pre-school and school-going children (n = 550, 1–14 years old) from the rural areas of the state of Sikkim—an NE Indian state, with respect to associated demographic factors was investigated. A total of 550 fecal *E*. *coli* isolates were collected during July 2015 to June 2017. A structured questionnaire was used to collect data to ascertain the potential factors associated with the carriage of antibiotic resistance *E*. *coli* among the children. Statistical analysis along with a logistic regression identified potential external factors affecting the observed antibiotic resistance pattern. The data indicated a high prevalence of resistance to common antibiotics like ampicillin (92%), ceftazidime (90%), cefoxitin (88%), streptomycin (40%) and tetracycline (36%), but no resistance to chloramphenicol. The resistance to the combination of penicillin and quinolone group of antibiotics was observed in fifty-two percent of the isolates. A positive correlation between the harboring of antibiotics resistant *E*. *coli* with different demographic factors was observed such as, with children living in nuclear family (*vs* joint family 63.15%, OR 0.18, 95% CI:0.11–0.28, p < 0.01), below higher secondary maternal education (*vs* college graduates 59.27% OR 0.75, 95% CI:0.55–1.02, p < 0.02). A close association between different demographic factors and the high prevalence of antibiotic-resistant commensal *E*. *coli* in the current study suggests a concern over rising misuse of antibiotics that warrants a future threat of emergence of multidrug-resistant pathogen isolates.

## Introduction

During the last decade, an alarming worldwide increase in the incidence of community-acquired infections with pathogens resistant to multiple antibiotics of common use has been observed [1]. Use of antibiotics plays a crucial role in the emergence of antibiotic resistance amongst pathogenic bacteria worldwide as well as in developing countries [2] [3] [4]. However, to counter the prevailing scenario, very few new antibiotics have been introduced in the last three decades [5]. Inappropriate use of antimicrobials is considered to be one of the main factors responsible for the high prevalence of antibiotic-resistant pathogens in developing countries [4]. Increased antibiotic resistance in pathogens leads to increased mortality and morbidity, enhanced transmission and increased associated health care costs [6].

A large number of bacteria colonize the gastrointestinal tract of mammals [7] as a part of normal commensal microflora. They play an important role in human nutrition and health by promoting nutrient supply, preventing pathogen colonization, shaping and maintaining the homeostasis of the intestinal immune system [6]. It has been observed that the use of antibiotics affects the composition and population of commensal bacteria harbored in the gut [4][8]. In community settings, young children tend to be the most exposed to antibiotics and several studies have indicated that the younger children have the highest risk of carrying antibiotic resistant commensal bacteria in communities [9][8][10].

*Escherichia coli* is a member of the *Enterobacteriaceae* family, the enteric bacteria, which are facultative anaerobic Gram-negative bacteria, commonly found in the intestinal tract of warm-blooded animals including humans [7]. *E*. *coli*, a near-ubiquitous colonizer of the gastrointestinal tract in children and adults has often been used in studies of the incidence of antibiotic resistance in commensal bacteria [11]. In recent years, the potential role of the commensal microbiota in the emergence and spread of antimicrobial resistance in pathogens has been universally acknowledged [12]. Some species of the commensal microbiota, such as fecal *E*. *coli*, have been exploited as a sensitive indicator for the surveillance and spread of antimicrobial resistance among pathogens [13].

In one of the study, it was reported that commensal *E*. *coli* isolates from neonates less than one month of age without any prior exposure to the antibiotics were highly resistant to individual antibiotics like ampicillin (100%) and co-trimoxazole (96%) [14]. Recently, Purohit *et al*., 2017 evaluated the prevalence of antibiotic resistance (ampicillin, cefoxitin, nalidixic acid, polymyxin-B etc.) in commensal *E*. *coli* isolates from human, animals, and water by disk diffusion method and reported that commensal *E*. *coli* from all sources displayed resistance to all the antibiotics tested except polymyxin-B [15]. They also reported a higher incidence of antibiotic resistance in human isolates as compared to that from environment or animals [15]. Similarly, Moran *et al*., 2017 have also observed the high prevalence of antibiotic resistance in the commensal *E*. *coli* of humans [16]. In one of the study, it was observed that adults carried more antibiotic-resistant commensal *E*. *coli* as compared to the young ones [17]. They report huge differences between resistance to at least one antimicrobial agent (75.7% *vs* 54.7%) as well as multi-drug resistance (30.4% *vs* 14%) between adult *vs* young ones [17]. Recent reports suggest an increase in the incidence of diseases by antibiotics resistance pathogens in the region. Gajamer *et al*., 2018 have reported that as a result of observed antibiotic resistance increase in the pathogens, imipenem and gentamycin are becoming the first choice of the drug against commonly isolated uropathogens [18]. In another study, a similar increase in the uropathogens antibiotic resistance against commonly used antibiotics like ciprofloxacin has been reported [19]. A study by us also revealed that commonly used antibiotics like penicillin, amoxicillin as well as third generation cephalosporins like ceftriaxone and cefuroxime may no longer be used as the first line of the drug against urinary tract infection [20].

India is among the nations with the highest burden of bacterial infections and the crude mortality from the infectious diseases is about 417 persons per 100,000 [21]. In 2010, India was the world’s largest consumer of antibiotics for human health with 12.9 x 10^9^ units of antibiotic consumption (~10.7 units per person) [2]. Global antibiotic consumption index was reportedly high among the BRICS countries, *i*.*e*., Brazil, Russia, India, China, and South Africa during the period 2000–2010. Among the BRICS nations, 23% of the retail antibiotics sales was attributable to India [22]. Antimicrobial resistance in pathogens is a major public health concern in India. The emergence of antimicrobial resistance is not only limited to the older and more frequently used classes of drugs but to the newer and more expensive drugs such as carbapenem as well [21]. Extended-spectrum beta-lactamase (ESBL) producing strains of *Enterobacteriaceae* have emerged as a big challenge in the hospitalized patients as well as in the community where *E*. *coli* isolates as high as 61% have been found to be the ESBL producers [21].

Limited research has been done with regards to prevalence of antibiotic resistance in *E*. *coli* isolates among Indian children [8][23][24]. However, none of these studies were done in the northeastern population, hence there is no data suggesting the prevalence of antibiotic resistance in this area. In the few studies conducted, the wide variation has been demonstrated in resistance pattern of *E*. *coli* isolates from reportedly healthy children [14][17] [8]. To evaluate the correlation between increasing antibiotic use and the emergence of antibiotic-resistant pathogens, community-based surveillance could be of great use [8]. The aim of the present study was to investigate the prevalence of antibiotic resistance in commensal *E*. *coli* harbored by children in the hills of northeastern Himalayan regions and to identify various demographic factors associated with its carriage or spread in the children.

## Materials and methods

### Site and study duration

The selected study area was the Sikkim state of India and the study was conducted from July 2015 to June 2017. Sikkim is a northeastern state of India surrounded by Tibetan Plateaus in North, Chumbi Valley of Tibet as well as Bhutan kingdom in East. In the South, it is surrounded by Darjeeling district of West Bengal state of India and in the West, it borders with Nepal [25]. Sikkim has a total population of 6,10,577 in which 4, 56,999 (74.85%) live in rural areas [26]. In Sikkim, the literacy rate is 81.4%. The literacy rate is higher in males (86.6%) as compared to females (75.6%) [27]. The land of Sikkim is generally divided into lower hills (altitude 270 to 1500 meters), middle hills (altitude 1500 to 2000 meters) and higher hills (altitude 2000 to 3000 meters) based on the geographical parameter. The upper reaches are divided into an alpine zone (altitude above 3900 to 5000 meters with vegetation) and snowbound land (very high mountains without vegetation up to 8580 meters) [25].

### Survey

**Fig 1:**
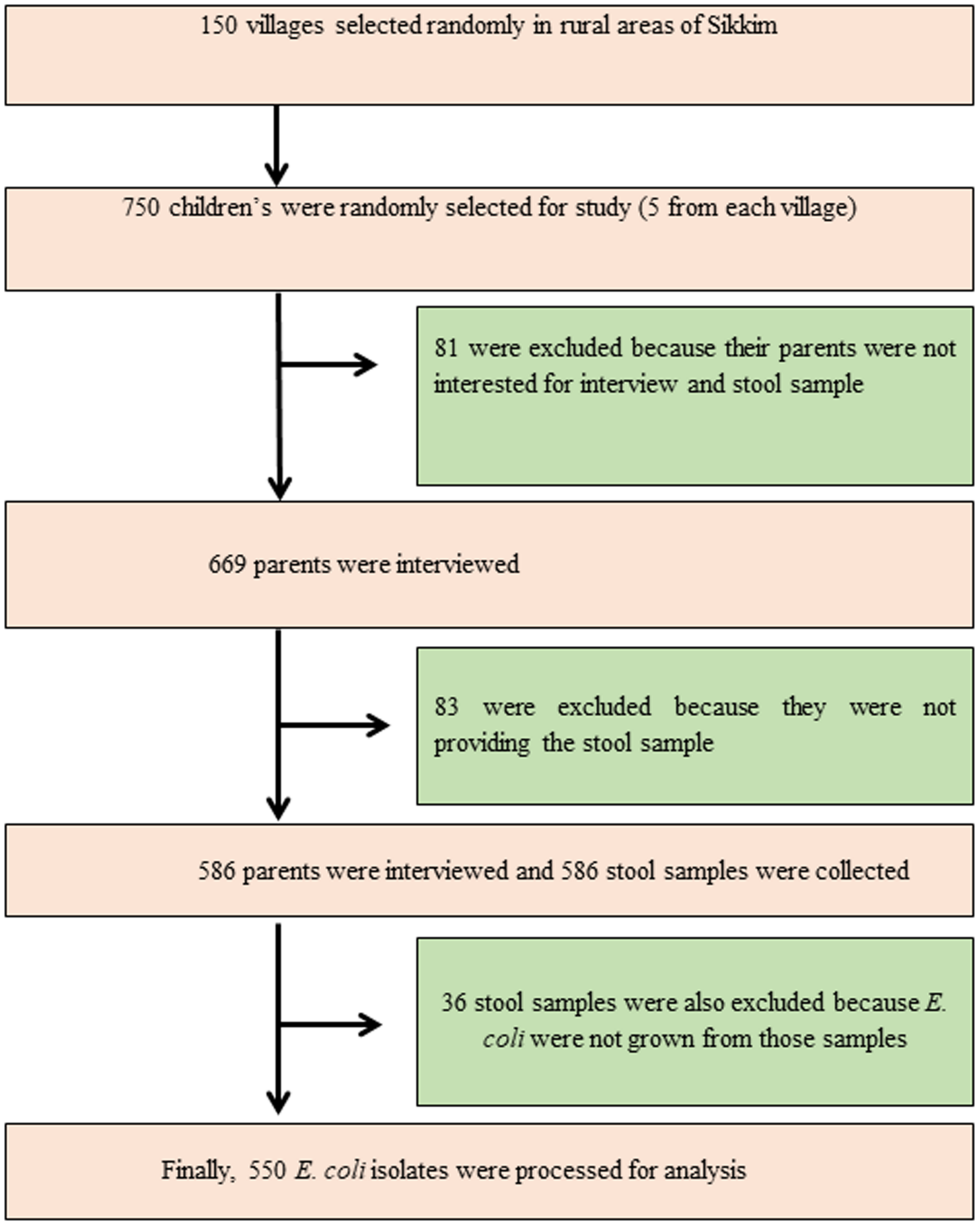
Study design. Informed written consent from parents/guardians were procured on the proforma (S1 File). Total 586 stool samples were collected from children of age group 1–14 years. Of the 586 stool samples, 36 stool samples were excluded as no definitive *E*. *coli* isolates could be cultured from those samples. A total of 550 *E*. *coli* isolates from 550 samples were used for the analysis.

**Fig 2:**
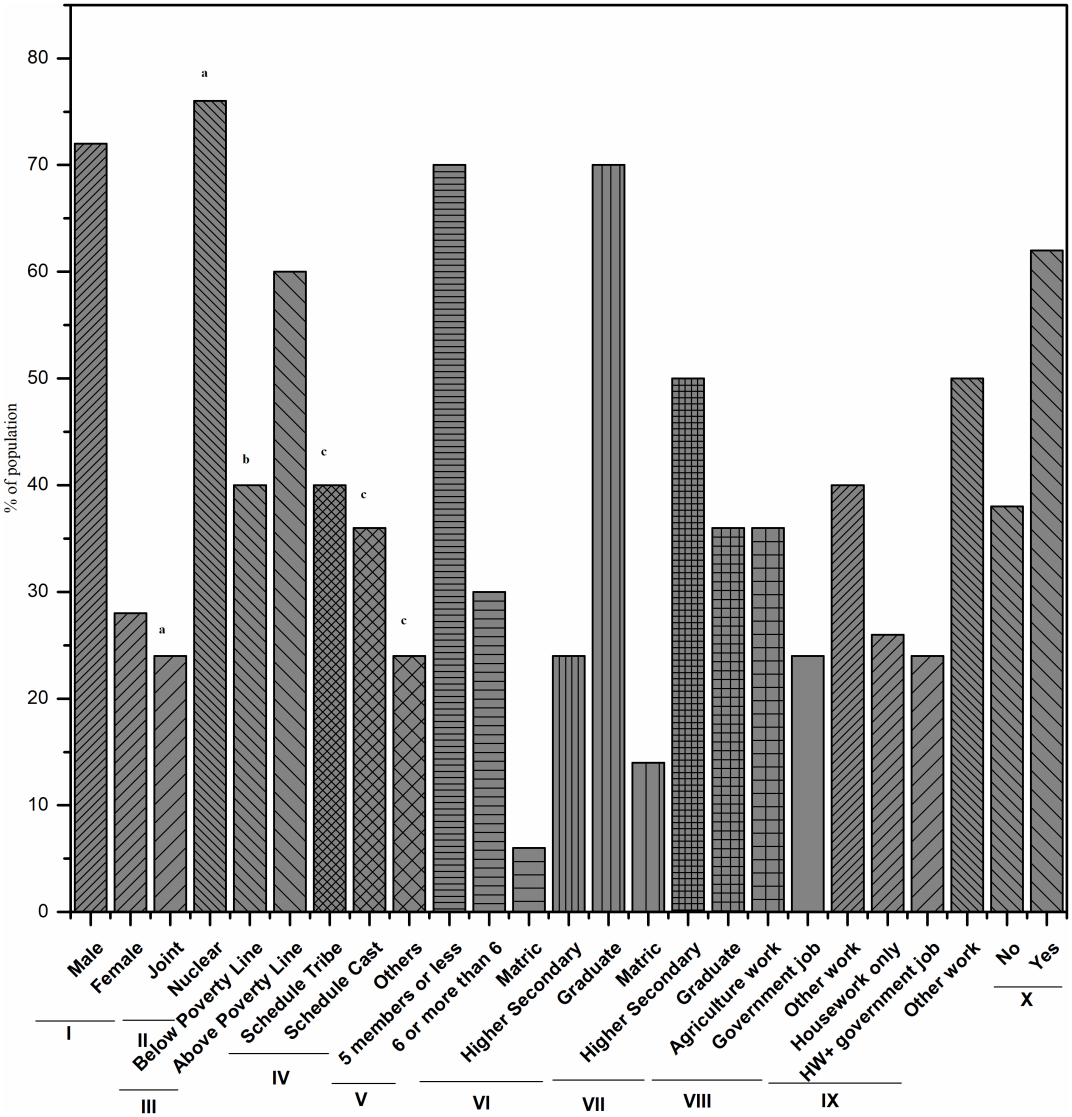
Demographic details of community participants. Demographic details of community participants and their families are represented as a percentage of the population against the total population (N = 550). a: ‘Nuclear family’ referred to those families having only parents and children living in one household premises and ‘Joint family’ referred to the families having parents, children and their relatives living in one household premises; b = Family below poverty line refers to those families having possession of “Below Poverty Line (BPL)’ card issued by Government of India (GOI); c = Scheduled castes, backward castes and scheduled tribes are special status groups of socially and economically deprived classes of people as defined by the GOI; I: Gender of Children; II: Family type; III: Economic status; IV: Caste; V: Number of family members; VI: Paternal education; VII: Maternal education; VIII: Maternal occupation; IX: Antibiotics used last month.

Sikkim has a total of 451 villages with a total rural population of 4,56,999 as per census 2011 [27]. The survey was carried out in randomly selected 150 villages of Sikkim at different altitude. The study design and the demographic details of the respondent were summarized and presented in Figs 1 and 2. The Gram Panchayat Units (GPU) were communicated and given prior information about the survey to be conducted. Only healthy children from the age group of 1–14 years were included in this study while ‘unhealthy children’ were excluded. The 'healthy children’ group included metabolically active children that did not present any symptomatic diseases, especially gastrointestinal abnormalities like diarrhea, vomiting, abdominal cramps, nausea at the time of survey while the ‘unhealthy children’ group included children that displayed any kind of gastrointestinal abnormalities at the time of the survey. The minimum required sample size (n = 350) was calculated using the OpenEpi software version 3.0.1 with finite population correlation (FPC) factor for 95% confidence interval with the frequency of resistant to at least one drug was 50% [28]. Prior to the study, informed written consent was obtained from the parents/guardians (S1 File). The resistance to even a single antibiotic is a cause for concern as it highlights the problem of antibiotic resistance prevalence and the potential reservoir of genes available to pathogens for developing antibiotic resistance. The structured questionnaires were used to interview the parents for making the decision about the inclusion of their wards in the study (S2 File). The parents with their wards were invited to participate in the study, with an assurance that they can withdraw from the study with all their details if at any point they experienced any difficulty or developed apprehensions regarding the survey.

### Fecal sample collection

During the survey, families were provided with clean, sterile, wide mouth, screw-capped specimen container for stool collection. Parents/guardians were asked to collect morning stool for the study [26]. All the stool samples were transported to the laboratory in cool condition on the ice and processed within 6 hours of collection. Samples were streaked on MacConkey agar (Hi-Media) and Eosin Methylene Blue (EMB) agar plates (Hi-media) for the isolation of *E*. *coli*. The isolates were further confirmed by phenotypic characterization and standard biochemical tests as reported previously [26].

The confirmed *E*. *coli* were further subjected to antibiotic susceptibility test by standard Kirby-Bauer disc diffusion method using CLSI guideline (Clinical and Laboratory Standards Institute, 2014) [29]. The antibiotics discs impregnated with an indicated concentration of particular antibiotics were used. The antibiotic impregnated discs used in the study were ampicillin (30 mcg, cat no. SD077, Hi-media), amoxicillin (30 mcg, cat no. SD0076, Hi-media), ciprofloxacin (5 mcg, cat no. SD060, Hi-media), norfloxacin (10 mcg, cat no. SD057, Hi-media), cefoxitin (30 mcg, cat no. SD041, Hi-media), ceftazidime (30 mcg, cat no. SD062, Hi-media), imipenem (10 mcg, cat no. SD073, Hi-media), chloramphenicol (30 mcg, cat no. SD006, Hi-media), streptomycin (10 mcg, cat no. SD031, Hi-media), tetracycline (30 mcg, cat no. SD037, Hi-media), netillin (30 mcg, cat no. SD046, Hi-media), polymyxin-B (300 U, cat no. SD029, Hi-media), ofloxacin (5 mcg, cat no. SD087, Hi-media) and amikacin (30 mcg, cat no. SD035, Hi-media).

A standard culture of *E*. *coli* (MTCC 10898) was used as antibiotic sensitive control culture with each batch of antimicrobial susceptibility test. The isolates that displayed resistance to three different groups of antibiotics were designated as multi-drug resistant (MDR) bacteria as indicated above. Extended-Spectrum ß-Lactamases test was also performed using ESBL Kit (Hi-Media, Mumbai, India). The *Klebsiella pneumoniae* (MTCC 9024) was used as a positive control for ESBL detection.

### Statistical analysis

Drug susceptibility data and filled questionnaires were collected and entered in XL-STAT. The response of the participants provided on the structured questionnaires were analyzed as described previously [8]. Briefly, data were analyzed using descriptive statistics, bivariate analysis (cross-tabulation) and frequency distribution. The statistical significance was assumed at the p ≤ 0.05 (5% significance). The association between socio-demographic parameters and health behavior of participants was correlated with the laboratory outcomes of the antibiotic resistance displayed by *E*. *coli* isolates. The association between the variables was tested using Chi-Square test. Those variables which approached statistical significance at p < 0.2 were entered into the multivariate logistic regression model. The odds ratio, confidence interval and correlation between the antibiotic resistance pattern and the demographic structure (cluster analysis) were analyzed using R statistics (ver. 3.4, package = ggplot and epiR).

## Results

The demographic details of the families of 550 children from whom *E*. *coli* were isolated are summarized in Fig 2. The median age of children included in the study was 8.5 years for males and 8 years for females. Twenty-six percent (n = 143) of the children had a history of gastrointestinal illness in the last three weeks prior to the survey and they were on antibiotics. Among the 550 *E*. *coli* isolates from 550 children, 90% percent (n = 495) were found to be resistant to at least one of the commonly used antibiotics (ADR). Multidrug resistance (MDR) was displayed by > one-fourth of the isolates (41%, n = 226) while only 3% of the isolates (n = 17) were found to be ESBL producers.

**Fig 3:**
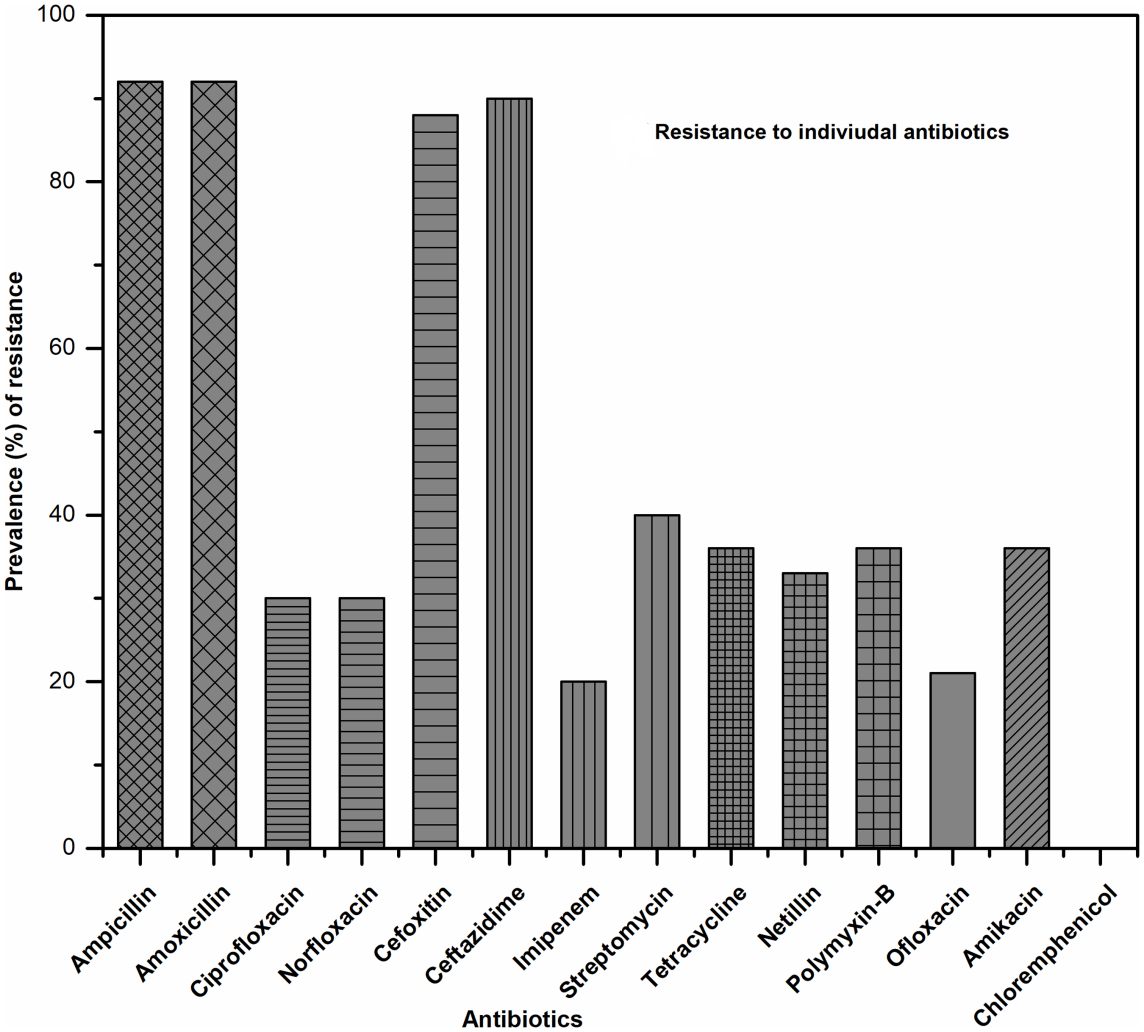
Resistance pattern of *E*. *coli* isolates against individual antibiotics.

**Fig 4:**
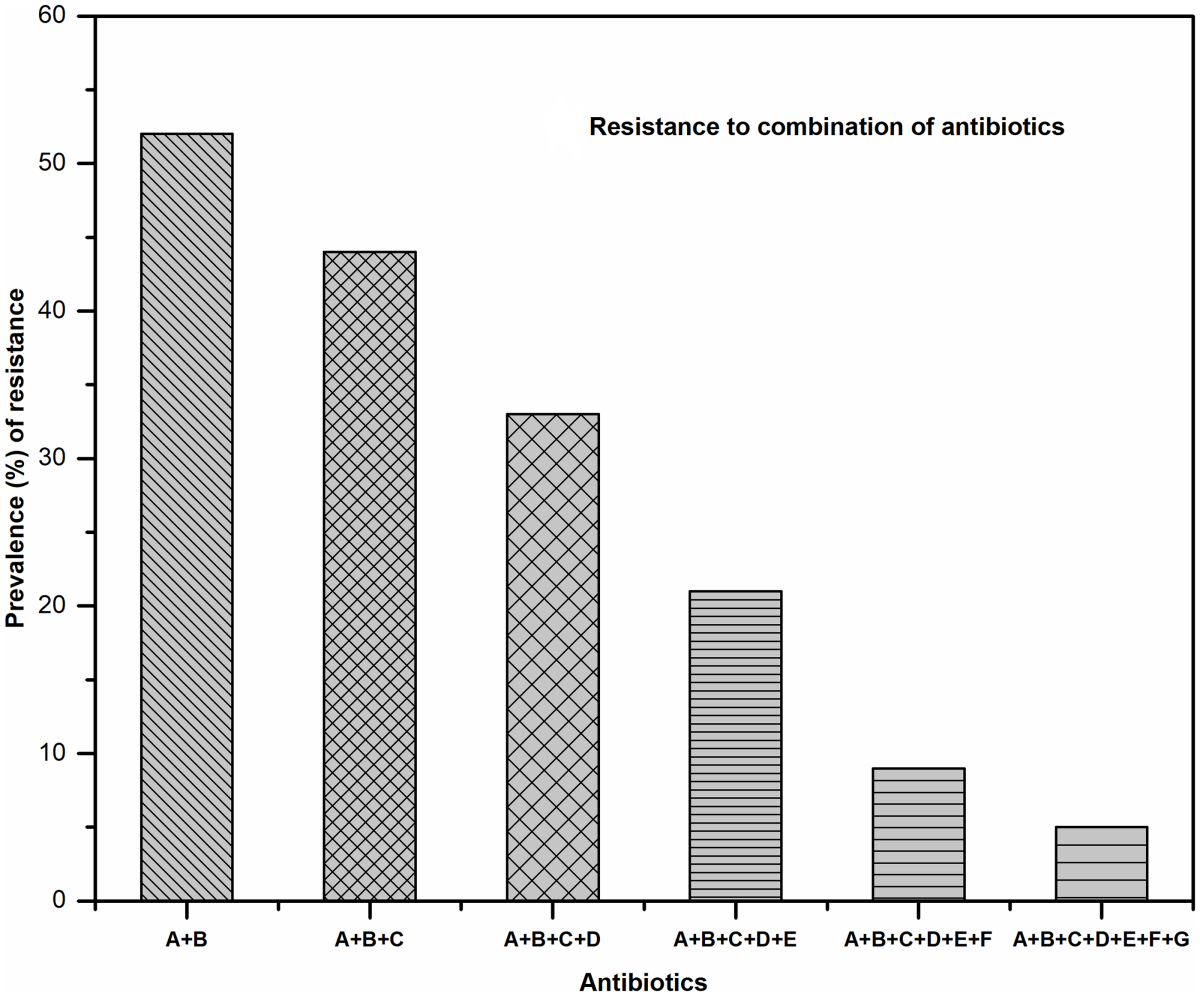
Resistance pattern of *E*. *coli* isolates against a combination of antibiotics. (Group A = Penicillin, B = Quinolones/Fluoroquinolones, C = Cephalosporin, D = Carbapenem, E = Aminoglycosides, F = Tetracycline, G = Polypeptide).

**Fig 5:**
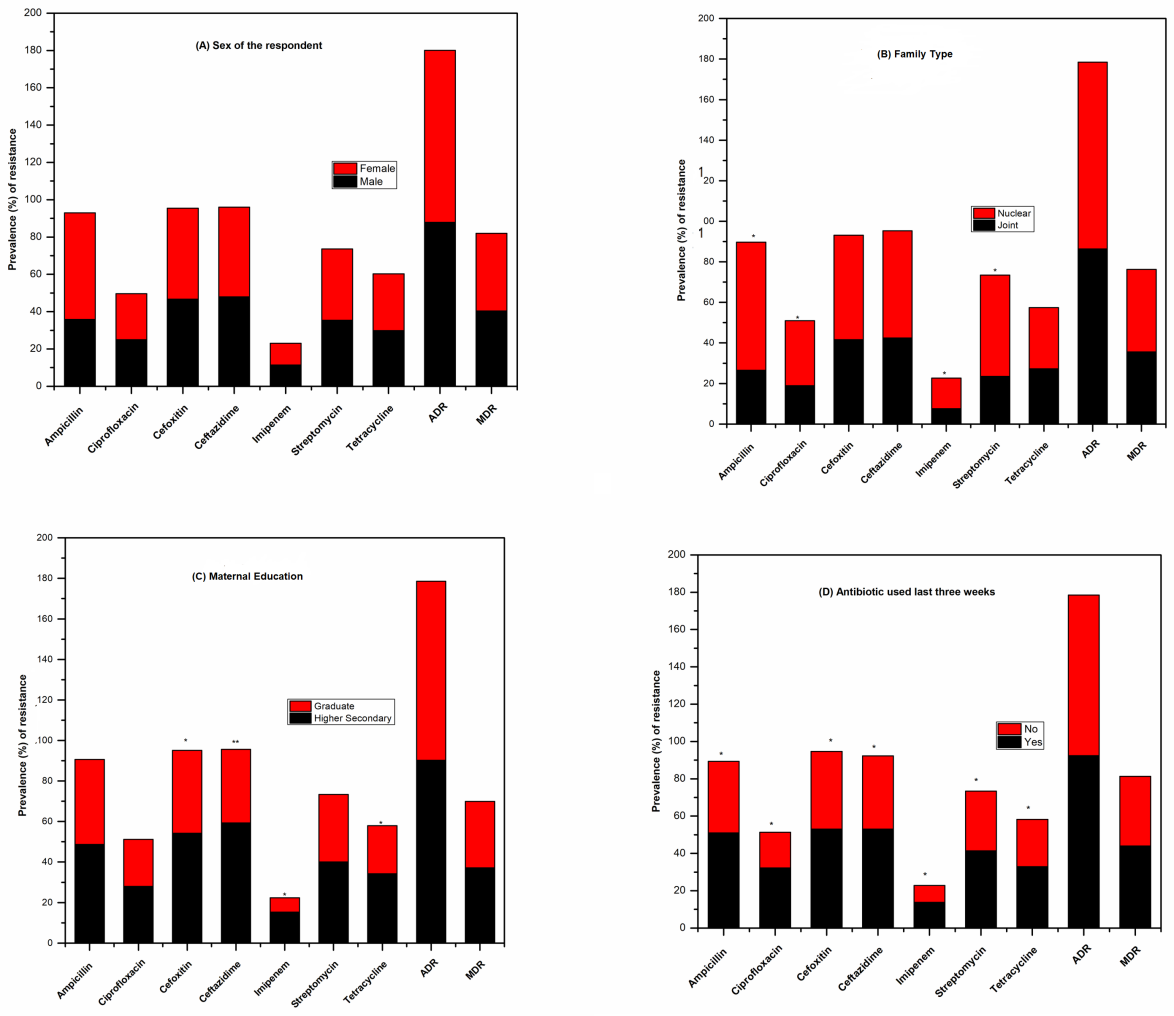
Association of the antibiotic resistance prevalence (%) in *E*. *coli* with demographic variables. The data displays the observed antibiotic resistance in *E*. *coli* isolates from, (A) male and female, (B) children living in joint and nuclear families, (C) children with their mother having education up to graduate and higher secondary level and (D) children with prior antibiotic exposures. The potential association between demographic variables and antibiotic resistance pattern observed for the isolates was evaluated using chi-square analysis and multivariate logistic regression. *Statistically significant by chi-square analysis (p < 0.01); ** statistically significant by multivariate logistic regression.

The resistance to single antibiotics (Fig 3) and a combination of antibiotics (Fig 4) shared a correlation with the demographic variables (Fig 5). Commensal *E*. *coli* displayed a similar pattern of resistance to all antibiotics tested in both males and females except for ampicillin. The number of isolates showing resistance to ampicillin was higher in case of female children (57.14%) as compared to male (35.85%) but it was statistically non-significant. As compared to isolates from children living in joint families, a higher percentage of isolates from children living in nuclear families were found to be resistant to ampicillin (OR 0.18, 95% CI: 0.11–0.28, p < 0.01), ciprofloxacin (OR 0.48, 95% CI: 0.28–0.80, p < 0.01), imipenem (OR 0.28, 95% CI: 0.10–0.80, p ≤ 0.01), and streptomycin (OR 0.31, 95% CI: 0.20–0.50, p < 0.01). Lesser number of *E*. *coli* isolates from children whose mothers were college graduates displayed resistance to cefoxitin (OR 1.69, 95% CI: 1.16–2.46, p < 0.01), ceftazidime (OR 0.75, 95% CI: 0.55–1.02, p < 0.02), imipenem (OR 3.24, 95% CI: 1.46–7.17, p < 0.01), and tetracycline (OR 1.75, 95% CI: 1.12–2.74, p < 0.01) as compared to those from children of higher secondary school educated mothers. Paternal education was seemingly not associated with the antibiotic resistance pattern of *E*. *coli* isolates from children.

The percentage of *E*. *coli* isolates displaying ADR varied widely from 30 to 90% among different villages and that for MDR varied from 10 to 41% (Fig 5). Children on antibiotics with a history of gastrointestinal illness (last three weeks) had a high rate of carriage of *E*. *coli* with ADR and MDR in comparison to children without any history of gastrointestinal illness and antibiotic use (Fig 5). However, the observed differential carriage of ADR and MDR *E*. *coli* isolates by children based on their antibiotics usage history were statistically not significant. Interestingly, children who had received antibiotics in last three weeks had odds of carrying *E*. *coli* isolates which were resistant to all the antibiotics as compared to children who were not exposed to antibiotics (p < 0.01). The age of children had no association with the occurrence of ADR or MDR *E*. *coli* isolate. Other demographic variables like economic status, caste, number of family members, paternal education, paternal occupation, and maternal occupation were not associated with the isolation of antibiotic resistant *E*. *coli* isolates.

**Fig 6:**
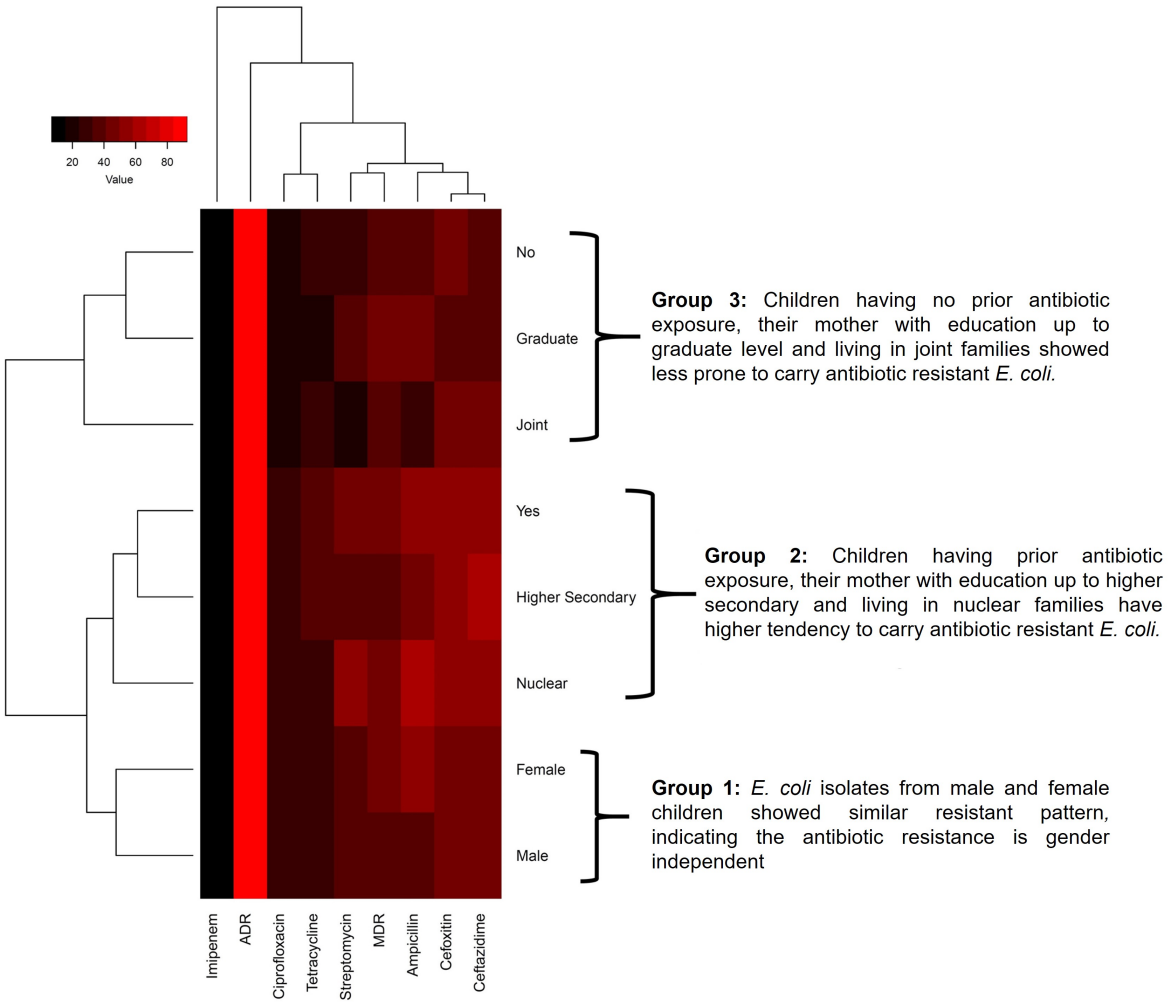
Cluster analysis representing the relationship between demographic factors and pattern of antibiotic resistance. It formed three distinct groups/clusters based on the antibiotic resistance pattern. Group 1 represents the *E*. *coli* isolates from male and female which showed similar resistance pattern. Group 2 represents the demographic factors which make children more prone to carry antibiotic-resistant commensal *E*. *coli*. Group 3 represents the demographic factor associated with least antibiotic resistance.

In cluster analysis (Software: R Statistics, Package: ggplot, Function: heatmap.2), the demographic data formed three distinct groups based on the observed antibiotic resistance pattern of *E*. *coli* isolates. The first group was formed by the *E*. *coli* isolates of male and female children. Though they apparently differed, it was statistically not significant (Fig 5), suggesting the antibiotic resistance in commensal *E*. *coli* in our study population to be gender independent. The second group was formed by *E*. *coli* isolates from the children living in nuclear families having prior exposure to antibiotics and children of school-dropout mothers displaying highest antibiotic resistance occurrence. The third group was formed by the children from the mothers who were graduates, without any prior antibiotic consumption history before the study (last three weeks) and children living in a joint family. The third group was the carrier of least number of antibiotic resistant *E*. *coli* isolates (Fig 6). Thus, analysis of the cluster pattern showed a clear demographic association of antibiotic resistance in *E*. *coli* isolates. Antibiotic resistance pattern displayed by the isolates was independent of the gender of the children while it was highly dependent on the mother’s education and the type of family to which the children belonged. Children of college graduate mothers and living in the joint family were less likely to carry antibiotic resistant *E*. *coli* than those of higher secondary school graduate mothers and living in nuclear families.

## Discussion

This is the first community-based study of its own kind that explored the prevalence of antibiotic-resistant commensal *E*. *coli* in the children of rural areas of Sikkim as an indication or indicator of the emergence of antibiotic resistance. Antibiotic resistance (AR) has become one of the world’s most pressing public health problems of this era. Unnecessary use of antibiotics without prescription for treatment of common bacterial infection is considered as one of the leading causes of the emergence of antibiotic resistance in common pathogens. The AR can make easily treatable illness a dangerous infection leading to prolonged sufferings. It can spread to the family members, peer groups, and community through different means. Shared distribution of *E*. *coli* in the lower abdomen of human and its susceptible nature to the frequently used antibiotics makes it a good indicator bacterium to study the potential spread and emergence of antibiotic resistance. The current study was designed to assess the prevalence and distribution of antibiotic resistance in *E*. *coli* isolated from the stool samples of the healthy children of Sikkim, a northeastern state of India. The results were correspondingly correlated with the demographic data to ascertain the effect of community structure on antibiotic resistance pattern of commensal *E*. *coli*.

### Antibiotic-resistance pattern observed in *E*. *coli* isolates from children

In this study, 90% of the isolated *E*. *coli* from the healthy children showed ADR property, *i*.*e*., resistant to at least one antibiotic. This occurrence rate is much higher than the previously reported rates in *E*. *coli* isolates from Central (72%), Eastern (38–68%) and Southern (63%) India [8][30][24]. A study conducted in Greece in 1998 reported that about 40% of healthy children carried *E*. *coli* which were resistant to at least one antibiotic tested [30]. The study showed that healthy children carried commensal *E*. *coli* with higher incidence of resistance to the first generation of antibiotics (common antibiotics) like ampicillin (92%), streptomycin (40%), and tetracycline (36%). Correlative results were also reported from Peru and Bolivia where higher resistance was observed against common antibiotics (95%) [13]. One of the interesting finds of the current study was that in contrast to Peru and Bolivia where 70% of the isolates displayed chloramphenicol resistance, no such resistance to chloramphenicol was observed in *E*. *coli* isolates from Sikkim [13]. The probable reason behind the chloramphenicol susceptibility of the isolates may be the result of infrequent consumption of chloramphenicol in the region as this antibiotic is prescribed for diseases like typhoid, cholera, conjunctivitis *etc*. which are less prevalent in Sikkim. The possible reason behind the development of resistance to the common antibiotics could be the frequent use or misuse of the antibiotics due to easy availability and affordability [8].

In Sikkim, 41% of the *E*. *coli* isolates showed MDR which is much higher than MDR reported from the eastern region of India. In the eastern region of India, Odisha had recorded 24% incidence of MDR in *E*. *coli*, while the central part of India, Ujjain (largest city of Madhya Pradesh) had recorded 33% of MDR in *E*. *coli* isolates [8][30]. Southern India had historically recorded significantly higher percentage of commensal MDR *E*. *coli* (42%) than the eastern and middle India, but it is reciprocal to the current study (41%) conducted in Sikkim [23]. The observed increase in the incidence of ADR and MDR in *E*. *coli* isolates in the study area possibly reflects the recent spread and emergence of the antibiotic resistance gene pool in general and commensal *E*. *coli* in particular. This resistant pattern is a matter of concern as it is directly correlated with the health index of children of the community [31].

### Demographic factors influence the antibiotics resistance pattern of *E*. *coli*

Demographic structure of a community is generally held as a key factor for the emergence and outbreak of antibiotic resistance in bacteria. Children’s age, sex, parental education, socioeconomic status, family type and frequency of antibiotics taken are some of the important demographic parameters which were considered in the current study. During the study, we did not find any indication of the effect of age on the antibiotic resistance (age: 1–14) as previously reported [24][30][8][4][13]. There are only a few reports available about the effect of children’s gender on the prevalence of antibiotic resistance. Some of the earlier studies had reported that the male children harbor more antibiotic resistant *E*. *coli* isolates as compared to female children [30][13][4], whereas some others had observed the opposite [10][8]. We also observed that the antibiotic resistance pattern of *E*. *coli* isolates was independent of the children’s gender, similar to the findings reported previously [8]. Statistically, the resistance pattern observed among the isolates from male and female children were not different (p < 0.01).

Maternal education as a demographic factor is supposed to play an important role in the development of antibiotic resistance in host microbiota and pathogens [32][8]. Some of the earlier studies have reported an association between maternal education and the level of antibiotic resistance observed [8][33][34][35]. In the current study, a correlation between mothers’ education status and the incidence of ADR *E*. *coli* isolate in children was observed. It was found that children whose mothers were college graduates were less prone to carry antibiotic-resistant commensal *E*. *coli* than whose mothers were only higher secondary school graduates (Fig 5). The isolates of *E*. *coli* from mothers having education up to graduate level were found to be less resistant to antibiotics like cefoxitin (OR 1.69, 95% CI: 1.16–2.46, p < 0.01), ceftazidime (OR 0.75, 95% CI: 0.55–1.02, p < 0.02), imipenem (OR 3.24, 95% CI: 1.46–7.17, p < 0.01) and tetracycline (OR 1.75, 95% CI: 1.12–2.74, p < 0.01). The plausible correlative reason could be the knowledge and awareness of the more educated mothers about the proper use of different antibiotics and the doses required. Often it has been observed that the mothers with higher secondary education or less are not that much aware of the suggested dose proposition and the guidelines of antibiotics use. They are also less likely to follow the prescribed course and doses of antibiotics suggested by the physician. Hence, they are often prone to committing the antibiotic misuse. The children living in joint family were also found to be less prone to carrying the resistant isolates of *E*. *coli* as compared to the nuclear families. As for a reason, it could be due to the availability of proper medical guidance of the elderly in the families on antibiotic consumption which is not possible in nuclear families where the parents are busier with their chores and duties.

Geographically, the land of Sikkim host mountain ranges with varied altitude (350 -8500m). In this study, the data indicate that the distance between market and villages and the altitude of a village play an important role in the development of antibiotic resistance in *E*. *coli*. The samples from villages near a market or town area were found to have a higher prevalence of antibiotic-resistant *E*. *coli* compared to the villages which were farther away. A similar pattern was also observed for the villages situated at higher altitudes where a higher prevalence of antibiotic-resistant *E*. *coli* correlated with the easier access to a pharmacy. A study carried out by Shakya *et al*., (2013) in Ujjain, India, made similar observations in which more antibiotic resistant *E*. *coli* isolates were found in people living nearby or those having easy access to pharmacies to get antibiotics. [8]. Besides above stated contributory factors, environmental factors are also supposed to play an important role in the variation in the incidence rates of antibiotic resistant *E*. *coli* isolates between villages [3]. In the rural areas of Sikkim, water is supplied directly from springs (surface water) to community outlets without any treatment. There is no centralized water supply system. So, exposure to the environmental contaminants may create variation in the water supply from village to village. The human effluent and agricultural runoff containing the antibiotic-resistant bacteria can act as contributors to the development of antibiotic resistance in commensal bacteria [36][3]. The current study only considered the antibiotic resistance pattern displayed by commensal *E*. *coli* in the rural communities of the hills of Sikkim. Though a large number of antibiotics were used in this study to evaluate the emerging antibiotic resistance, we only used one isolate from each child. So, there exists a chance that some of the resistant isolates might have been missed out and the estimated prevalence of antibiotic resistance might be an underestimate of the problem.

## Conclusion

The current study identified a high prevalence of antibiotic-resistant commensal *E*. *coli* in children of rural areas of Sikkim, a state in Northeastern India. The antibiotics resistance pattern was evaluated for individual antibiotics and also for the combination of two to three classes of antibiotics. There was a significant level of correlation between the prevalence of antibiotic resistance in commensal *E*. *coli* isolates from the children and the demographic variables like mother’s education, type of family and the access and use of antibiotics. However, the prevalence of antibiotic resistance in *E*. *coli* isolates did not display any association with child’s gender. The recovery of a significantly high number of ADR and MDR isolates from the children of the region warrants an immediate need for the commitment to ensure the rational use of antibiotics as much as possible. This is the first report from hills of the eastern Himalayan region regarding the antibiotic resistance profile of gut *E*. *coli* isolates from children. The study suggests looming threat of the antibiotic-resistant pathogens to the area and mandates immediate counteractive measures including educating the community about the proper uses of antibiotics and appropriate hygiene habits to tackle the growing problem. This study warrants further investigation into the problem encompassing broader adjoining areas of South East Asia that share the geography and have similar livelihood practices to design a comprehensive strategy for the containment of the problem in the region.

## Supporting information

S1 File Informed written consent form in two languages (Nepali and English). (PDF)

S2 File Format of questionnaire used during the study. (DOCX)
