# Supplementary material for: Prevalence of antibiotic resistance in commensal Escherichia coli among the children in rural hill communities of Northeast India
Source: PLoS One. 2018 Jun 18;13(6):e0199179. doi: 10.1371/journal.pone.0199179 (PMC6005495; doi:10.1371/journal.pone.0199179)
Supplement: S1 File — (PDF) [file pone.0199179.s001.pdf]

सहमति फारम

(यो फारम सहभागी/मातापिता/अभिभावक तथा शोधार्थीले भर्नु पर्नेछ)

म आफ्ना छोरा/ छोरी/ पति वा पत्नीलाई सँगै राखेर यस शोध कार्य, विषय "Microbial Study of Spring Water in Sikkim" मा एकजना सहभागी भएर कार्य गर्न सहमत छु।

यस अध्ययनमा मेरा व्यक्तिगत जानकारी एवं दिशाको नमूनाहरू सङ्ग्रह गरिने छन्।

सहभागीको रूपमा मलाई बुझाइएका अनि मैले बुझेअनुसार यो अध्ययन वैज्ञानिक शोधको निम्ति महत्वपूर्ण छ अनि यसले मेरो स्वस्थ्य/सामाजिक स्तरमा कुनै प्रतिकूल असर पार्दैन।

सहभागी/मातापिता/अभिभावकको नाम एवं हस्ताक्षर:

मातापिता वा अभिभावक भए सहभागीसितको सम्बन्ध

दिनाङ्क:

शोधार्थीको हस्ताक्षर

Consent Form

(To be filled by the participants/parents/guardians and researcher)

I along with my son/daughter/spouse do hereby agree to be a participant in the research work as title **"Microbial Study of Spring Water in Sikkim"**

In this study my personal information and **Stool** sample (s) will be collected.

As a participant, I have been explained and have understood that this study is important for scientific research and has no adverse effect on my health/social status.

Name & Signature of the participant/ parents/ guardian:

Relation with participant in case of parents/guardians etc:

Date: Signature of Researcher
