# Supplementary material for: Prevalence of antibiotic resistance in commensal Escherichia coli among the children in rural hill communities of Northeast India
Source: PLoS One. 2018 Jun 18;13(6):e0199179. doi: 10.1371/journal.pone.0199179 (PMC6005495; doi:10.1371/journal.pone.0199179)
Supplement: S2 File — (DOCX) [file pone.0199179.s002.docx]

**Study of Prevalence of Antibiotic Resistance in commensal *E. coli* in hill community of North East India**

| Demographics |
| --- |
| 1. Gender- Male/Female |
| 1. Age (yrs.)-(1-6)/ (7-14) |
| 1. Population type- a. Rural b. Semi-rural c. Urban |
| 1. Name of Place------------- |
| 1. Name of Village: |
| 1. District- North/west/East/West |

| Family Type:   1. Joint b. Nuclear |
| --- |
| Economic Status:   1. Below Poverty Line 2. Above Poverty Line |
| Cast:   1. Schedule Cast 2. Schedule Tribe 3. Others |
| Number of Family Members:   1. 5 member or less 2. 6 or more than 6   Paternal Education:   1. Matric 2. Higher Secondary 3. Graduate |
| Maternal Education:   1. Matric 2. Higher Secondary 3. Graduate |
| Paternal Occupation:   1. Agriculture work 2. Government Job 3. Other Work |
| Maternal Occupation:   1. Housework only 2. Housework + Government Job 3. Other Work |
| Antibiotic Used last Month:   1. Yes 2. No |

| General information  Frequency of diet intake-   1. Twice b. Thrice |
| --- |
| Use of Antibiotics-   1. Never b. Occasional c. Regular |
| Major Dietary composition-   1. Vegitarian b. Non-vegitarian c. Vegan |
| Diet Type-   1. Cocked b. Un-cooked c. Boiled d. Steamed e. Canned |
| Food Source-   1. Own Farm b. Market c. Both |
| Consumption of Fermented Food-   1. Yes b. No |
| Fermented Food type-   1. Gundaruk b. Kinema c. Churpi d. Dahi e. Dry Meat |
|  |

| Social/life style behavior |
| --- |
| Smoking-   1. Yes 2. No |
| Alcohol consumption-   1. Yes 2. No |
| Frequency of alcohol consumption-   1. Daily 2. Weekly 3. Occasionally |
| Preferred alcoholic beverage-   1. Wine 2. Brandy 3. Beer 4. Rum 5. Whisky 6. Home brew |
| Appropriate sanitary condition-   1. Yes 2. No |
| Cooking infrastructure-   1. Hygienic 2. Un-hygienic |
